# Supplementary material for: Modeling glioblastoma heterogeneity as a dynamic network of cell states
Source: Mol Syst Biol. 2021 Sep 16;17(9):e10105. doi: 10.15252/msb.202010105 (PMC8444284; doi:10.15252/msb.202010105)
Supplement: Supplementary file 5 — Source Data for Figure 3 [file MSB-17-e10105-s001.zip › Figure3A_sourcedata/GSEA_3065/nosigdb_state4.GseaPreranked.1622718430994/NB_KI_CLUSTER5.html]

Details for gene set NB\_KI\_CLUSTER5[GSEA]

|  || Dataset | state4 |
| Phenotype | NoPhenotypeAvailable |
| Upregulated in class | na\_neg |
| GeneSet | NB\_KI\_CLUSTER5 |
| Enrichment Score (ES) | -0.8834685 |
| Normalized Enrichment Score (NES) | -3.1043 |
| Nominal p-value | 0.0 |
| FDR q-value | 0.0 |
| FWER p-Value | 0.0 |
Table: GSEA Results Summary

  

Fig 1: Enrichment plot: NB\_KI\_CLUSTER5      
 Profile of the Running ES Score & Positions of GeneSet Members on the Rank Ordered List

  

| PROBE | GENE SYMBOL | GENE\_TITLE | RANK IN GENE LIST | RANK METRIC SCORE | RUNNING ES | CORE ENRICHMENT || 1 | GAPDH |  |  | 494 | 0.138 | -0.0752 | No |
| 2 | TUBA1B |  |  | 3658 | 0.007 | -0.5869 | No |
| 3 | CDKN2D |  |  | 4933 | -0.077 | -0.7904 | No |
| 4 | RHEB |  |  | 5346 | -0.113 | -0.8532 | No |
| 5 | H2AFV |  |  | 5381 | -0.117 | -0.8547 | No |
| 6 | SPDL1 |  |  | 5560 | -0.137 | -0.8788 | Yes |
| 7 | BUB3 |  |  | 5583 | -0.141 | -0.8775 | Yes |
| 8 | CENPW |  |  | 5585 | -0.142 | -0.8728 | Yes |
| 9 | TEX30 |  |  | 5629 | -0.148 | -0.8746 | Yes |
| 10 | CEP152 |  |  | 5649 | -0.152 | -0.8725 | Yes |
| 11 | HIST2H2AC |  |  | 5670 | -0.155 | -0.8704 | Yes |
| 12 | NUCKS1 |  |  | 5724 | -0.166 | -0.8733 | Yes |
| 13 | NCAPD2 |  |  | 5778 | -0.179 | -0.8757 | Yes |
| 14 | TRIP13 |  |  | 5792 | -0.181 | -0.8716 | Yes |
| 15 | TCF19 |  |  | 5796 | -0.182 | -0.8658 | Yes |
| 16 | ZWILCH |  |  | 5805 | -0.184 | -0.8608 | Yes |
| 17 | PRC1 |  |  | 5835 | -0.192 | -0.8589 | Yes |
| 18 | PLK1 |  |  | 5836 | -0.192 | -0.8523 | Yes |
| 19 | CDCA7 |  |  | 5863 | -0.198 | -0.8497 | Yes |
| 20 | HIST1H1E |  |  | 5865 | -0.198 | -0.8431 | Yes |
| 21 | DDX11 |  |  | 5867 | -0.198 | -0.8364 | Yes |
| 22 | CKAP2 |  |  | 5879 | -0.201 | -0.8313 | Yes |
| 23 | ANP32B |  |  | 5890 | -0.204 | -0.8259 | Yes |
| 24 | DIAPH3 |  |  | 5893 | -0.205 | -0.8192 | Yes |
| 25 | PRR11 |  |  | 5926 | -0.214 | -0.8170 | Yes |
| 26 | RANBP1 |  |  | 5939 | -0.218 | -0.8114 | Yes |
| 27 | RACGAP1 |  |  | 5953 | -0.223 | -0.8059 | Yes |
| 28 | CIP2A |  |  | 5956 | -0.225 | -0.7985 | Yes |
| 29 | MZT1 |  |  | 5989 | -0.235 | -0.7956 | Yes |
| 30 | RAN |  |  | 5990 | -0.235 | -0.7875 | Yes |
| 31 | LIG1 |  |  | 6002 | -0.239 | -0.7811 | Yes |
| 32 | CHEK1 |  |  | 6025 | -0.248 | -0.7761 | Yes |
| 33 | MXD3 |  |  | 6042 | -0.265 | -0.7696 | Yes |
| 34 | HMGB1 |  |  | 6047 | -0.267 | -0.7610 | Yes |
| 35 | MCM5 |  |  | 6069 | -0.278 | -0.7549 | Yes |
| 36 | SHCBP1 |  |  | 6071 | -0.279 | -0.7455 | Yes |
| 37 | HIST1H1C |  |  | 6077 | -0.284 | -0.7365 | Yes |
| 38 | GMNN |  |  | 6080 | -0.285 | -0.7270 | Yes |
| 39 | FBXO5 |  |  | 6084 | -0.287 | -0.7177 | Yes |
| 40 | DEK |  |  | 6092 | -0.292 | -0.7088 | Yes |
| 41 | FANCA |  |  | 6097 | -0.295 | -0.6993 | Yes |
| 42 | CDT1 |  |  | 6098 | -0.295 | -0.6891 | Yes |
| 43 | DUT |  |  | 6107 | -0.303 | -0.6800 | Yes |
| 44 | MCM3 |  |  | 6113 | -0.307 | -0.6703 | Yes |
| 45 | LBR |  |  | 6118 | -0.312 | -0.6602 | Yes |
| 46 | MIS18BP1 |  |  | 6122 | -0.316 | -0.6498 | Yes |
| 47 | ARL6IP1 |  |  | 6145 | -0.336 | -0.6419 | Yes |
| 48 | NUDT1 |  |  | 6146 | -0.338 | -0.6303 | Yes |
| 49 | EZH2 |  |  | 6159 | -0.348 | -0.6202 | Yes |
| 50 | HMGN2 |  |  | 6163 | -0.351 | -0.6087 | Yes |
| 51 | ANP32E |  |  | 6168 | -0.357 | -0.5970 | Yes |
| 52 | CENPK |  |  | 6169 | -0.363 | -0.5845 | Yes |
| 53 | KIF20B |  |  | 6172 | -0.373 | -0.5721 | Yes |
| 54 | PCNA |  |  | 6173 | -0.374 | -0.5592 | Yes |
| 55 | ECT2 |  |  | 6178 | -0.382 | -0.5467 | Yes |
| 56 | CENPM |  |  | 6179 | -0.386 | -0.5335 | Yes |
| 57 | DBF4 |  |  | 6180 | -0.387 | -0.5202 | Yes |
| 58 | DNMT1 |  |  | 6182 | -0.396 | -0.5067 | Yes |
| 59 | CHAF1A |  |  | 6183 | -0.396 | -0.4931 | Yes |
| 60 | NASP |  |  | 6187 | -0.403 | -0.4797 | Yes |
| 61 | SGO2 |  |  | 6192 | -0.413 | -0.4662 | Yes |
| 62 | GINS2 |  |  | 6194 | -0.420 | -0.4519 | Yes |
| 63 | ZWINT |  |  | 6195 | -0.425 | -0.4373 | Yes |
| 64 | TUBB4B |  |  | 6196 | -0.431 | -0.4225 | Yes |
| 65 | BRCA1 |  |  | 6197 | -0.435 | -0.4075 | Yes |
| 66 | DHFR |  |  | 6200 | -0.441 | -0.3927 | Yes |
| 67 | BIRC5 |  |  | 6214 | -0.484 | -0.3782 | Yes |
| 68 | CLSPN |  |  | 6218 | -0.490 | -0.3618 | Yes |
| 69 | SMC4 |  |  | 6219 | -0.490 | -0.3449 | Yes |
| 70 | HELLS |  |  | 6221 | -0.501 | -0.3279 | Yes |
| 71 | SMC2 |  |  | 6229 | -0.518 | -0.3112 | Yes |
| 72 | GGH |  |  | 6232 | -0.522 | -0.2936 | Yes |
| 73 | CKS1B |  |  | 6234 | -0.523 | -0.2758 | Yes |
| 74 | KPNA2 |  |  | 6236 | -0.543 | -0.2573 | Yes |
| 75 | CKS2 |  |  | 6239 | -0.585 | -0.2375 | Yes |
| 76 | UBE2T |  |  | 6240 | -0.593 | -0.2171 | Yes |
| 77 | ATAD2 |  |  | 6243 | -0.607 | -0.1966 | Yes |
| 78 | UBE2S |  |  | 6244 | -0.617 | -0.1753 | Yes |
| 79 | PTTG1 |  |  | 6245 | -0.622 | -0.1540 | Yes |
| 80 | CDK1 |  |  | 6246 | -0.627 | -0.1324 | Yes |
| 81 | H2AFZ |  |  | 6250 | -0.651 | -0.1106 | Yes |
| 82 | MCM7 |  |  | 6252 | -0.665 | -0.0879 | Yes |
| 83 | HMGB2 |  |  | 6258 | -0.766 | -0.0623 | Yes |
| 84 | PCLAF |  |  | 6259 | -0.790 | -0.0352 | Yes |
| 85 | HIST1H4C |  |  | 6262 | -1.038 | 0.0002 | Yes |
Table: GSEA details [plain text format]

  

Fig 2: NB\_KI\_CLUSTER5: Random ES distribution      
 Gene set null distribution of ES for **NB\_KI\_CLUSTER5**
